# Implementation, recruitment and baseline characteristics: A randomized trial of combined treatments for smoking cessation and weight control

**DOI:** 10.1016/j.conctc.2017.06.003

**Published:** 2017-06-15

**Authors:** Terry Bush, Jennifer Lovejoy, Harold Javitz, Stacey Mahuna, Alula Jimenez Torres, Ken Wassum, Brooke Magnusson, Cody Benedict, Bonnie Spring

**Affiliations:** a Alere Wellbeing, Solely Owned Subsidiary of Optum, 999 Third Avenue Suite 1800, Seattle, WA 98104-1139, USA; b Arivale, Inc and University of Washington School of Public Health, Seattle, WA, USA; c SRI International, Menlo Park, CA, USA; d Amazon, Seattle, WA, USA; e Formerly at Alere Wellbeing/Optum, Seattle, WA, USA; f Adara Development, Seattle, WA, USA; g Gates Foundation (previously at Alere Wellbeing), Seattle, WA, USA; h Center for Behavior and Health, Feinberg School of Medicine, Northwestern University, Chicago, IL, USA

**Keywords:** Smoking, Weight gain, Quitlines, Simultaneous, Sequential

## Abstract

**Background:**

Two-thirds of treatment-seeking smokers are obese or overweight. Most smokers are concerned about gaining weight after quitting. The average smoker experiences modest post-quit weight gain which discourages many smokers from quitting. Although evidence suggests that combined interventions to help smokers quit smoking and prevent weight gain can be helpful, studies have not been replicated in real world settings.

**Methods:**

This paper describes recruitment and participant characteristics of the Best Quit Study, a 3-arm randomized controlled trial testing tobacco cessation treatment alone or combined with simultaneous or sequential weight management. Study participants were recruited via tobacco quitlines from August 5, 2013 to December 15, 2014.

**Results:**

Statistical analysis on baseline data was conducted in 2015/2016. Among 5082 potentially eligible callers to a tobacco quitline, 2540 were randomized (50% of eligible). Compared with individuals eligible but not randomized, those randomized were significantly more likely to be female (65.7% vs 54.5%, p < 0.01), overweight or obese (76.3% vs 62.5%, p < 0.01), more confident in quitting (p < 0.01), more addicted (first cigarette within 5 min: 50.0% vs 44.4%, p < 0.01), and have a chronic disease (28.6% vs. 24.4%, p < 0.01). Randomized groups were not statistically significantly different on demographics, tobacco or weight variables. Two-thirds of participants were female and white with a mean age of 43.

**Conclusions:**

Adding weight management interventions to tobacco cessation quitlines was feasible and acceptable to smokers. If successful for cessation and weight outcomes, a combined intervention may provide a treatment approach for addressing weight gain with smoking cessation through tobacco quitlines.

**Trial registration:**

Clinicaltrials.gov NCT01867983.

## 1 Introduction

Tobacco use continues to incur high costs to individuals, families and many nations [1]. In 2014, 16.8% of U.S. adults (95% CI = 16.1–17.4) reported they were currently smoking and a majority expressed a desire to quit and have made at least one quit attempt [2]. Smoking cessation counseling and FDA approved medications help individuals quit tobacco but relapse is high [3]. Quit rates using point prevalence intent-to-treat abstinence for telephone based cessation treatments vary greatly depending on treatment intensity, survey response rates and demographic characteristics of callers. For example, published quit rates range from 14 to 50% at 6 months and 17–23% at 12 months [4], [5], [6], [7], [8], [9], [10]. Notably, the likelihood of gaining weight is cited as a common barrier to successful quitting [11], [12], [13], [14]. Research indicates that approximately 75% of smokers gain weight after quitting and that the weight gain is usually modest (5–6 kg) [15], [16], [17], [18], [19]. However, it is estimated that 10–15% of smokers who are trying to quit gain more than 10 kg and the weight gain can be permanent without lifestyle adjustments [20]. Efforts to curb excessive weight gain while maintaining a successful quit have led to the development and testing of smoking cessation interventions that also address weight gain [16], [17], [21], [22], [23]. Systematic reviews of such combined treatments have shown that some interventions that added weight control content to cessation treatment limited quit related weight gain, at least for the short-term, without harming smoking abstinence [16], [17], [21], [22], [24]. However, the latest Cochrane review concluded that evidence was insufficient to make strong recommendations for adding weight based interventions to tobacco cessation treatment [16]. Given this uncertainty, more research is needed. One successful study by Spring and colleagues compared tobacco cessation treatment alone versus the simultaneous or sequential addition of a weight management intervention [25]. Results showed that a sequential treatment approach (weight management treatment after the quit date), reduced weight gain to a greater extent than simultaneous treatment or cessation treatment alone [25]. Surprisingly, both combined treatments showed a non-significant trend for better cessation rates than smoking cessation only. This trial was intensive, in-person, group-based and involved only women smokers, like most other prior intervention trials testing combined smoking and weight interventions. The Best Quit study (BQS), described in an earlier protocol paper [26], aims to replicate and extend this prior efficacy trial in the context of an effectiveness study [25]. To our knowledge no studies have tested combined tobacco and weight control interventions in a population based setting. The study protocol and interventions from the prior trial were adapted for telephone delivery and delivered via national tobacco cessation quitlines. Quitlines are an ideal setting for testing and disseminating successful interventions in part because, like the general population, over two thirds of state quitline callers are overweight or obese and two thirds have significant concerns about gaining weight after quitting tobacco [8].

This paper describes the acceptability of adding weight based content to state and commercial quitlines by describing recruitment challenges and enrolled participants.

## 2 Study objectives and results

This paper addresses the following questions: Will smokers seeking help via quitlines want help limiting weight gain? Will smokers accept the invitation to participate in the randomized trial? Are baseline characteristics similar across the three groups?

## 3 Materials and methods

The Best Quit Study (BQS) is a 3-arm randomized controlled trial in which eligible and consenting smokers who called a state or employer-sponsored quitline were randomly assigned to tobacco cessation treatment alone, to the simultaneous delivery of tobacco and weight management treatment, or to tobacco cessation treatment followed by weight management intervention. The study was approved by the Western Institutional Review Board and is described in detail in a prior paper [26].

### 3.1 Setting

This translational, effectiveness study was conducted at Alere Wellbeing (a solely owned subsidiary of Optum) which is the largest provider of tobacco cessation quitlines in the US, serving 27 States and 750 employer groups and health plans. We conducted the study in ten quitlines from nationally distributed employer groups (commercial quitlines) and three state quitlines (Indiana, Maryland, North Carolina).

### 3.2 Population

Participants who called into the quitline between August 5, 2013 and December 15, 2014 were eligible for the study if they were age 18 and older, smoked at least 10 cigarettes per day, stated that they were ready to quit in the next 30 days, requested counseling, and were able to speak and read English. Additional screening criteria were access to a phone and internet and willingness to receive 10 phone calls from the quitline. Exclusion criteria included pregnancy, a BMI below 18.5, prior or planned weight loss surgery, currently enrolled in a weight loss program, and having diabetes or a current eating disorder. The latter variable was assessed with one question: ‘Have you ever been told by a healthcare provider or mental health professional that you have an eating disorder such as anorexia or bulimia?’ We excluded underweight individuals (BMI < 18.5) because the weight management intervention was not designed for this population.

### 3.3 Recruitment and randomization

**Fig. 1:**
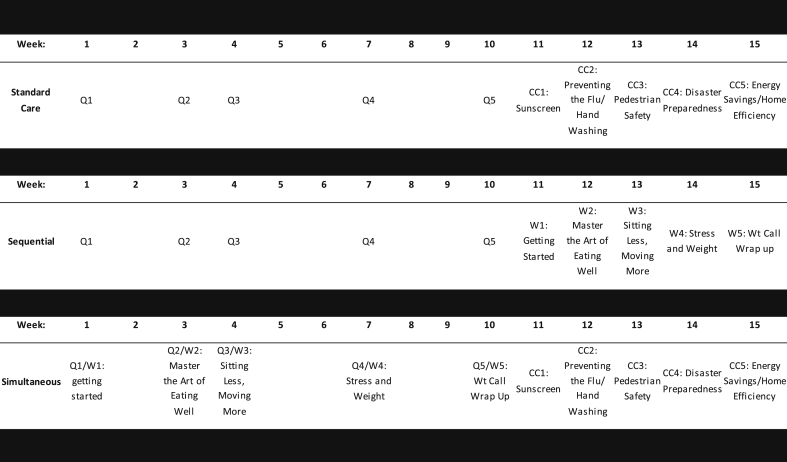
Call sequence for quit smoking (Q), weight management (W), and contact control (CC) calls.

Quitline callers who met eligibility criteria and gave informed consent to participate were randomly assigned in blocks of 10 without stratification by a computer generated algorithm to one of three groups of equal proportions: 1) tobacco cessation treatment, 2) simultaneous tobacco plus weight management treatment or 3) tobacco cessation followed by weight management (sequential treatment). All groups received 10 counseling calls. The first call was initiated by the tobacco user; the remaining calls were initiated by a coach. Participants were encouraged to call the quitline between proactive calls or after completing treatment if they wanted extra support. Because the standard tobacco cessation quitline offers 5 counseling calls and adding 5 weight management calls to the sequential group resulted in 10 calls, we created attention-matched conditions by adding 5 nonspecific healthy living calls to both the standard quitline protocol (contact control condition) and to the simultaneous treatment condition. In this way we were able to equalize the number of counseling sessions across the three groups (Fig. 1). To maximize participation rates in each call, coaches made 5 attempts to reach participants. Those not reached were sent reminder letters in the mail and an email stating ‘your coach is trying to reach you’.

### 3.4 Interventions

A comprehensive description of the interventions, counselor training and key intervention strategies are presented in a prior paper [26] and briefly summarized here. Alere Wellbeing Programs (Cessation treatment and Weight Management) are based on Social Cognitive Theory (SCT). Coaches use cognitive behavioral therapy (CBT), motivational interviewing (MI), modeling, reinforcement and principles of self-efficacy to achieve effective behavior change. Common counseling strategies include open-ended questions, reflections and strategies to elicit change talk, in which participants begin to articulate reasons why they should make healthy lifestyle choices.

The evidence based tobacco cessation treatment involved 5 coaching calls to help the smoker prepare for and successfully quit tobacco. Counseling calls were supplemented with mailed support materials and an interactive web-based program. Participants were also offered cessation medications in the form of nicotine patch, gum or lozenge (NRT) free of charge according to their state or employer plan benefits. In a standard 5-call program, counseling content and call timing was tailored to each person's availability to receive calls, their quit date and specific support requested. In general, call 1 involved assessment of their tobacco use and treatment needs, encouraging the use of NRT and setting a quit date. Call 2 supported a person near their quit date. Calls 3–5 provided ongoing support for successful tobacco cessation and plans for relapse prevention.

The evidence based weight management intervention, Weight Talk, involved 5 counseling calls, mailed materials and an interactive web-based program. For the purposes of this study, the weight-related component of the intervention aimed to prevent cessation-related weight gain, rather than to promote weight loss. Thus, the coaching goals were to reduce calories and increase physical activity sufficiently to offset metabolic slowing due to quitting smoking. Counseling content for each call is shown in Fig. 1. In call 2, Registered Dieticians (RDs) worked with participants to set an appropriate calorie reduction target. Coaches guided participants in ways to increase consumption of fruits and vegetables, reduce stress, and use the activity monitor to track and increase physical activity. Coaches educated participants about behavioral weight management techniques such as self-monitoring food intake and weight.

Healthy living counseling involved 5 calls covering health topics other than weight or tobacco. We chose to focus on sunscreen protection, flu protection, pedestrian safety, emergency preparedness and home energy savings. As shown in Fig. 1, for the standard tobacco group (contact control), coaches delivered 5 tobacco cessation calls (calls 1–5) followed by the 5 healthy living calls (calls 6–10). For the sequential group, coaches delivered 5 tobacco cessation calls first (calls 1–5) which were followed by 5 weight management calls (calls 6–10). For the simultaneous group, coaches delivered the combined intervention (tobacco treatment plus weight management) in calls 1–5 which were followed by the 5 healthy living calls (calls 6–10). The 10 coaching calls were intended to last 15–20 min each.

Bachelor or Masters level coaches were trained to deliver coaching in either tobacco, weight or both. The 2 weight groups received at least 1 call from an RD. Coaches were trained to follow a call protocol but the calls were not scripted. Calls were recorded for monitoring purposes and calls from approximately 10% of participants were coded to ensure treatment fidelity.

### 3.5 Measures

Survey data were self-reported at registration with the quitline and during the baseline interview prior to randomization. Data collected at registration are part of the minimum dataset recommended by the North American Quitline Consortium (NAQC) [27]. Demographic data included age, gender, ethnicity, race, marital status, and education level, current symptoms of depression and anxiety and presence of a chronic disease (Chronic Obstructive Pulmonary Disease (COPD), Coronary Artery Disease (CAD) or Asthma). We used the two item Patient Health Questionnaire (PHQ-2) as a measure of depression which has demonstrated good construct and criterion validity [28]. Tobacco related measures included number of cigarettes/day (CPD), time to first cigarette upon waking and exposure to smokers at home or work. We assessed motivation to quit with a single question: On a scale from 1 to 10, where 1 = not at all motivated and 10 = extremely motivated, how motivated are you to quit tobacco? We measured confidence in quitting with a single question: On a scale from 1 to 10, where 1 = not at all confident and 10 = extremely confident, how confident are you that you can quit tobacco? Weight related measures included height and weight, perceived expectation to gain weight (How likely do you think it is that you will gain weight as a result of quitting/staying quit using a 1–10 scale where 1 = not at all likely and 10 = extremely likely?), concern about weight gain (On a scale from 1 to 10, where 1 = not at all concerned and 10 = extremely concerned, how concerned are you about gaining weight as a result of quitting?), confidence in quitting without weight gain (On a scale from 1 to 10, where 1 = not at all confident and 10 = extremely confident, how confident are you that you can avoid gaining weight while staying quit?). The latter 3 questions were selected from two valid and reliable scales developed by Borrelli [13]. Body mass index (BMI) was calculated as the ratio of body weight to the body surface calculated using standard metric of kg/m^2^. Participants were classified as obese, overweight or normal weight according to standard BMI cut-points of greater than 30, 25–29.9, 18.5–24.9 respectively.

### 3.6 Data management

Registration data was collected by an ‘intake specialist’ when the participant called to enroll in the quitline. An automated system captured participant responses. Baseline data was collected by the study coach and participant responses were captured in the same automated system. Research staff monitored study enrollment and data collection throughout the study and provided analytic files at the end of recruitment.

### 3.7 Statistical analysis

Analysis took place in 2015–2016. Categorical variables were summarized by frequencies and percentages, and continuous variables were summarized by means and standard deviations. Pearson chi-square tests for categorical variables and *t*-tests for continuous variables were used to examine factors associated with enrollment in the study. Analyses of differences across the three randomized groups included Pearson chi-square for categorical variables and standard regression analyses for continuous variables. Given the number of comparisons made, we calculated false positive rates for the group.

## 4 Results

**Fig. 2:**
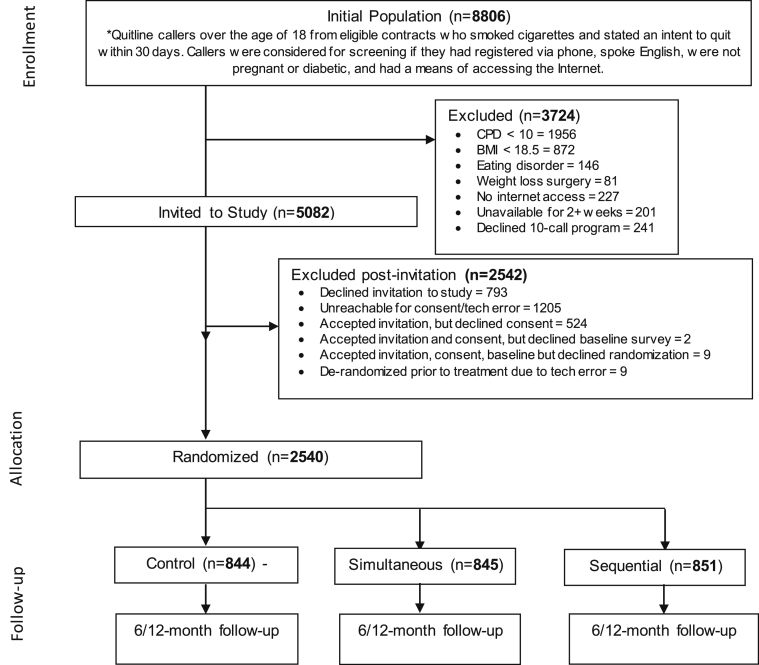
Best quit study CONSORT diagram.

Our goal was to recruit 2550 smokers in 18 months from commercially sponsored quitlines. Within three months, it became clear that the volume of calls from our 10 employer groups was insufficient to meet our target. Therefore, we added 3 state quitlines and met our goals within the timeline. Among the 8806 adult smokers who phoned one of the participating quitlines, 5082 (57.7%) were potentially eligible and invited to the study. Primary reasons for being ineligible (n = 3724) were smoking less than 10 CPD (n = 1956), having a BMI < 18.5 (n = 872), current eating disorder (n = 146), prior or planned weight loss surgery (n = 81), having no internet access (n = 227), being unavailable for the next 2 weeks (n = 201) or being uninterested in a 10-call program (n = 241). Callers who accepted the study invitation were transferred from the registration specialist to a study quit coach to complete their consent and baseline. If a coach was not immediately available, they were informed that a coach would call them within 24 h to complete enrollment and begin treatment. As a result of this delay, 1205 eligible participants were lost to follow up. The remainder who were not randomized included 793 who declined the study invitation, 526 who declined the consent or baseline survey, 9 who declined randomization and 9 who were de-randomized due to a technical error. Among the 5082 eligible callers, 3084 were contacted, 2558 completed the consent and baseline and 2540 (50% of eligible) were randomized (844 to Control; 851 to Sequential; 845 to Simultaneous). See consort diagram in Fig. 2.

As shown in Table 1, among the 5082 eligible smokers, those randomized (versus those who were not randomized), were more likely to be state quitline participants rather than commercial quitline participants (84.5% vs. 81.6%, p = 0.005), female (65.7% vs 54.5%, p < 0.001), have a chronic disease (28.6% vs. 24.4%, p < 0.001), be overweight or obese (76.3% vs 62.5%, p < 0.001), be more likely to smoke within 5 min of waking (p < 0.01) and smoke more CPD (p < 0.01). Groups did not differ on age, confidence in quitting or exposure to smokers at work or home.

**Table 1:** Eligible and randomized vs. Eligible but not randomized.^b^

Eligible	Randomized N = 2540	Eligible but Not randomized N = 2542^a^	Group comparison Statistic,p-value^a^	
State Quitlines Commercial Quitlines	84.5%	81.6%	χ^2^_(1)_ = 7.74, df = 5080, **p** = 0.005*	ES = 0.077
	15.5%	18.4%		
Gender (%female)	65.7%	54.5%	χ^2^_(1)_ = 67.1, df = 5079, **p** < 0.001*	ES = 0.230
Age: Mean (SD) Range	43.2 (12.2) 18–86	43.0 (12.6) 18–83	t = 0.63, df = 4902, p = 0.53	ES = 0.016
First cigarette within 5 min	50.0%	44.4%	χ^2^(1) = 9.70, df = 3581; **p** = **0.002**	ES = 0.112
Cigarettes/day: Mean (SD)	20.0 (8.3)	19.3 (8.0)	t = 3.1, df = 5079; **p** = 0.002*	ES = 0.086
Exposed to tobacco at home/work	60.3%	62.0%	χ^2^_(1)_ = 1.38, df = 4223, p = 0.24	ES = 0.035
Chronic Disease^c^ (Any of 4)	28.6%	24.4%	χ^2^_(1)_ = 13.9%, df = 5078, **p** < 0.001*	ES = 0.441
Confidence quitting: (1–10 scale) Mean (SD)	7.85 (2.1)	7.71 (2.1)	t = 1.89, df = 3447, p = 0.06	ES = 0.067
BMI Mean (SD), Range^d^	30.0 (7.11) 18.5–71.7	27.7 (6.23) 18.5–82.0	t = 11.9, df = 5080, **p** < 0.001*	ES = 0.344
% obese	43.6%	31.4%	χ^2^_(3)_ = 218.4, df = 5080, **p** < 0.001*	ES = 0.254
% overweight	32.7%	31.1%		ES = 0.034
% normal weight	23.7%	33.5%		ES = 0.218
% underweight	0.0%	4.0%		ES = 0.289

* Significant differences <.01.

a **Boldface** indicates statistical significance.

b Non-randomized group includes all those who were invited to the study but did not consent to baseline or randomization. Variables were collected during registration with the quitline.

c Asthma, COPD = chronic obstructive lung disease; CAD = coronary artery disease.

d Those who were obese were more likely to enroll and those of normal weight were less likely to enroll in the study.

As shown in Table 2, randomization was successful in yielding equal distributions across groups on demographic, tobacco and weight related characteristics. Overall, study participants were about 43 years of age, primarily female, overweight or obese, educated beyond high school, exposed to other smokers and highly motivated to quit smoking with moderate levels of confidence. Approximately a third reported they were currently “dieting.” A majority were enrolled in state quitlines and about 50% reported smoking a cigarette within 5 min of waking. Participant characteristics overall and by quitline (commercial or state) were similar to those found in other quitline studies and the general quitline population [29], [30], [31], [32], [33].

**Table 2:** Characteristics of randomized groups.^b^

Characteristic	Control N = 844	Sequential N = 851	Simultaneous N = 845	Total N = 2540	Group comparison^b^ Statistic, p-value^a^	Effect Size
State vs. Commercial quitlines	84.1% 15.9	84.1% 15.9%	85.3% 14.7%	84.5% 15.5%	χ^2^_(2)_ = 0.62, p = 0.74	ES = 0.0002
Medicaid	23.3%	24.9%	21.7%	23.3%	χ^2^_(2)_ = 2.51, p = 0.28	ES = 0.0004
Gender (% female)	66.7%	66.5%	64.0%	65.8%	χ^2^_(2)_ = 1.68, p = 0.432	ES = 0.0007
Age in years: Mean (SD) Range	43.2 (12.1) 18–72	43.0 (12.5) 18–86	43.5 (12.1) 18–77	43.2 (12.2), 18–86	F (2,2473) = 0.27; p = 0.766	ES = 0.0002
BMI: Mean (SD) Range	30.2 (7.2) 18.5–62.8	29.8 (7.0) 18.8–65.5	29.9 (7.1) 18.6–71.7	30.0 (7.1) 18.4–71.7	F (2,2537) = 0.81; p = 0.45	ES = 0.0006
% obese	43.7	44.1%	43.1%	43.6%	χ^2^_(4)_ = 3.43, p = 0.49	ES < 0.0001
% overweight	34.5%	31.1%	32.5%	32.7%		ES = 0.0009
% normal weight	21.8%	24.8%	24.4%	23.7%		ES = 0.0001
Marital status (% married)	33.3%	31.2%	33.1%	32.6%	χ^2^_(2)_ = 1.00, p = 0.61	ES = 0.0004
**Race**^c^						
% African American	25.3%	25.7%	28.3%	26.4%	χ^2^_(4)_ = 3.74, p = 0.442	ES = 0.0009
% White	69.0%	69.7%	66.0%	68.2%		ES = 0.0013
% other	5.6%	4.6%	5.7%	5.3%		ES = 0.0005
Ethnicity (% Hispanic)	2.4%	2.4%	2.1%	2.2%	χ^2^_(2)_ = 0.13, p = 0.94	ES < 0.001
Education beyond high school degree	57.4%	58.6%	55.5%	57.2%	χ^2^_(2)_ = 1.7, p = 0.43	ES = 0.0007
**Chronic Disease**^d^						
Asthma	17.1%	16.6%	16.5%	16.7%	χ^2^_(2)_ = 0.08, p = 0.96	ES < 0.0001
CAD	5.7%	5.5%	5.6%	5.6%	χ^2^_(2)_ = 0.03, p = 0.99	ES < 0.0001
COPD	14.4%	14.1%	15.3%	14.6%	χ^2^_(2)_ = 0.55, p = 0.77	ES = 0.0002
Diabetes (ineligible)	0.12	0.24	0.0	0.12%	χ^2^_(2)_ = 1.98, p = 0.37	ES = 0.0008
Chronic Disease (any of four)	29.1%	28.0%	28.8%	28.6%	χ^2^_(2)_ = 0.30, p = 0.86	ES = 0.0011
Exposed to smokers at home or work	59.8%	60.3%	60.7%	60.3%	χ^2^_(2)_ = 0.11, p = 0.95	ES = 0.0001
Cigarettes per day: Mean (SD) Range	19.7 (7.9) 10–44	20.0 (8.2) 10–44	20.4 (8.7) 10–44	20.0 (8.3) 10–44	F (2,2537) = 1.76, p = 0.17	ES = 0.0012
First cigarette within 5 min	50.5%	48.7%	50.9%	50.0%	χ^2^_(2)_ = 0.91, p = 0.64	ES = 0.0001
Quit motivation (1–10) Mean (SD)	8.9 (1.5)	9.0 (1.5)	8.9 (1.5)	8.9 (1.5)	F (2, 1887) = 0.47, p = 0.47	ES = 0.0006
Quit confidence (1–10);	7.9 (2.1)	7.9 (2.1)	7.8 (2.1)	7.8 (2.1)	F (2,2387) = 0.08; p = 0.92	ES = 0.0001
Past week days strenuous-moderate physical activity: Mean (SD) Range	2.5 (2.5) 0–7	2.5 (2.6) 0–7	2.5 (2.6) 0–7	2.5 (2.5) 0–7	F (2,2424) = 0.08; p = 0.92	ES < 0.0001
Past two weeks feeling anxious nearly every day	24% N = 833	25% N = 842	27% N = 832	25% N = 2507	χ^2^_(2)_ = 4.5, p = 0.61	ES = 0.0009
Anxiety level: (0–3 scale) Mean (SD)	1.27 (1.2)	1.30 (1.2)	1.32 (1.2)	1.30 (1.2)	F (2, 2504) = 0.41; p = 0.67	ES = 0.0004
Past two weeks feeling worried nearly every day	24%	23%	22%	23%	χ^2^_(2)_ = 0.38, p = 0.83	ES = 0.0001
Worry level: (0–3 scale) Mean (SD)	1.28 (1.14)	1.24 (1.16)	1.22 (1.15)	1.25 (1.15)	F (2,2508) = 0.44, p = 0.65	ES = 0.0003
Past two weeks loss of interest nearly every day,	13%	12%	13%	13%	χ^2^_(2)_ = 0.89, p = 0.64	ES = 0.0004
Loss of interest level: (0–3 scale) Mean (SD)	0.91 (1.0)	0.89 (1.0)	0.93 (1.0)	0.91 (1.0)	F (2,2478) = 0.26, p = 0.77	ES = 0.0003
Past two weeks depressive symptoms nearly every day	11%	10%	12%	11%	χ^2^_(2)_ = 0.96, p = 0.62	ES = 0.0004
Depression level: (0–3 scale), Mean (SD)	0.76 (0.99)	0.77 (0.98)	0.81 (1.0)	0.78 (1.0)	F (2,2505) = 0.55, p = 0.57	ES = 0.0005
Weight concerns (1–10) Mean (SD)	6.4 (3.1)	6.6 (3.0)	6.5 (3.1)	6.5 (3.1)	F (2,2527) = 0.79, p = 0.45	ES = 0.0007
Confidence in quitting without weight gain (1–10) Mean (SD)	5.7 (2.5)	5.6 (2.5)	5.5 (2.5)	5.6 (2.5)	F (2,2493) = 1.21, p = 0.30	ES = 0.0008
Perceived likelihood of weight gain (1–10) Mean (SD)	6.0 (2.8)	6.4 (2.8))	6.2 (2.9))	6.2 (2.8)	F (2,2382) = 4.06, **p** = **0.02**	ES = 0.0034
% Dieting to lose or maintain weight	33.3%	28.6%	32.9%	31.6%	χ^2^_(2)_ = 5.0, p = 0.08	ES = 0.0019
Expectation that treatment will help them quit smoking (1–10) Mean (SD)	8.4 (1.9)	8.3 (1.9)	8.4 (1.9)	8.4 (1.9)	F (2,2375) = 1.52, p = 0.22	ES = 0.0012

a Boldface indicates statistical significance.

b All analyses are for the full group but tabulations are for those with answers. Additional variables that are not present in Table 1 come from the baseline assessment.

c Race/ethnicity: Hispanic N = 58; White, non-Hispanic N = 1692; Black, non-Hispanic, N = 658; other, N = 132.

d Chronic Disease = Coronary Artery Disease, Chronic obstructive pulmonary disease or emphysema and/or Asthma.

## 5 Discussion

The main finding from this set of analyses was that nearly one third of adult smokers seeking help to quit via national quitlines (or 50% of those meeting the study eligibility criteria) were interested in participating in a study to help manage weight gain during tobacco cessation. In our study, we included both genders (41% were male) as well as individuals with low levels of weight concerns. Our rationale for not limiting the trial to women or those with weight concerns was that cessation related weight gain and concerns about weight gain are common for men and women across the BMI spectrum [8], [34]. Hence, smokers who are interested in combination tobacco and weight treatments may benefit from such treatment regardless of level of weight concerns [18]. Thus, we chose a more gender inclusive enrollment approach than that described by Spring and colleagues [25]. Another key finding is that two thirds of our study population self-reported they were dieting to maintain or lose weight. On average, participants expected to gain weight when they quit but were fairly confident they could avoid gaining weight after quitting.

Our goal was to test the feasibility of this approach in a setting in which the intervention, if successful in achieving the desired improvement in cessation and weight suppression, could be immediately disseminated and widely distributed. We did not, therefore, attempt to run an efficacy trial within this setting by introducing a waiting period prior to enrollment. Doing so would have resulted in the enrollment of a population more likely to be reached for follow-up calls, thereby reducing the loss to follow-up.

### 5.1 Potential limitations

We found that the approach of adding weight management to tobacco cessation was feasible to administer in a quitline setting and attracted smokers to sign up for this study. However, results only apply to those who could speak English as this was an inclusion criterion. Another limitation is that 1205 smokers who were invited to the study were unreachable for the consent and baseline assessment and therefore were not included in the study population. Also, although our original intention was to test the intervention within an employed population (commercial quitlines), the call volumes in our 10 participating commercial quitlines was insufficient to recruit the large numbers needed for the trial. Adding state quitlines enabled us to meet our recruitment goals on time, but sampled a more prevalent real-world population of smokers characterized by lower SES (fewer employed, fewer with medical insurance, fewer educated beyond high school), a higher prevalence of obesity and higher number of cigarettes per day. Ultimately, about 16% of study participants were recruited from commercial quitlines and 84% from state quitlines. State quitlines typically promote their tobacco cessation services to uninsured or underinsured participants. With the exception of Maryland, which offers medications to all participants regardless of insurance status, state participants must typically be uninsured or Medicare/Medicaid recipients in order to qualify for enhanced NRT benefits. State benefits can vary according to state and county and often do not provide the full recommended regimen of 8 weeks of NRT due to budget limitations. In contrast, smokers recruited from employer groups and health plans, must all be insured to participate in their sponsored tobacco cessation program. Another potential limitation is that some of the measures we used were not tested for reliability and validity. For example, we used single items of validated measures rather than the full scale in order to reduce participant burden and shorten the time between enrolling in the quitline and beginning the intervention. Most of the items have been used in prior studies and are recommended by the North American Quitline Consortium (NAQC). Another limitation is that height and weight was based on self-report. People generally underestimate their weight across time points and underestimation is disproportionately greater among those who are overweight or obese [35]. Studies have shown strong correlations between measured and self-reported weight indicating that self-reported weight is an excellent approximation of actual weight across a population [36].

In conclusion, quitlines provide a natural lab for translation of new treatments into real world settings, and a majority of overweight/obese smokers calling a quitline were open to participating in a treatment addressing weight as well as smoking. Although challenges exist in recruiting commercial quitline sites (e.g. employer groups) and keeping participants engaged, the ability to successfully adapt demanding evidence-based interventions for population-level delivery could have a significant public health impact.

## Ethics approval and consent to participate

The Western Institutional Review Board and the State of Maryland Institutional Review Board – in accordance with United States legislation — gave approval for this study. Participant consent will be obtained verbally via phone and documented by trained staff. Both programs (Quit For Life^®^ and Weight Talk^®^) are overseen by a clinical team at Alere Wellbeing. Measures are taken to insure adequate recruitment of women and minorities. All study participants will be over the age of 18. Alere is a HIPAA covered entity and complies with all HIPAA regulations regarding data security. The trial was registered at www.clinicaltrials.gov (1R01DA031147).

## Consent for publication

Not applicable.

## Availability of data and materials

The datasets generated during and/or analyzed during the current study are not publicly available due to the confidential nature of the data, but are available from the corresponding author on reasonable request.

## Competing interests

The authors at Alere Wellbeing declare that they are employed by Alere Wellbeing and have no other competing interests.

The author at SRI International declares that he has no competing interests.

The Co-PI, Dr. Bonnie Spring, declares that she has no competing interests.

The Co-I, Jennifer Lovejoy, declares that she has no competing interests.

## Funding

The project described was supported by Award Number RO1DA031147 from the National Institute on Drug Abuse (NIDA). The content is solely the responsibility of the authors and does not necessarily represent the official views of the National Institutes of Health.

## Authors contribution

TB is the PI on the study. She collaborated with BS, JL and HJ on developing the trial design, the interventions and other aspects of the study. She drafted the manuscript, collected feedback and approval from all authors, and submitted the final version of the manuscript. BS is the Co-PI on this study, which represents an effort to translate and implement her prior efficacy trial of a similar treatment to increase its population reach. She collaborated on the translation and implementation of study protocols, developing the trial design and analytic protocol, collaborated on overseeing trial implementation, and reviewed and edited all versions of the paper. JL co-developed the Weight Talk^®^ intervention and worked with the study team to translate the more intensive program for delivery within a 5 call program. She contributed to this final version of the paper. HJ served as the biostatistician for the trial. He led or collaborated on study design and implementation, and the analytic plans. He contributed to drafts of the paper. BM, CB and AJT acted as grant managers and oversaw protocol development, training and implementation and administrative tasks. SM is the research assistant for the study and assisted in technological aspects of the study. KW collaborated on treatment integration, trainings, implementation and call monitoring. All authors read and contributed to the writing of this paper.

## List of abbreviations

CI: confidence interval
Kg: kilogram
U.S.: United States
CPD: cigarettes per day
BMI: body mass index
NRT: nicotine replacement therapy
NIH: National Institutes of Health
SD: Standard deviation
FDA: Food and Drug Administration

## References

[1] (2014). The Health Consequences of Smoking: 50 Years of Progress: A Preport of the Surgeon General.

[2] Jamal A., Homa D.M., O'Connor E., Babb S.D., Caraballo R.S., Singh T. (2015). Current cigarette smoking among adults - United States, 2005-2014. MMWR Morb. Mortal. Wkly. Rep..

[3] Fiore M.C., Jaen R.C., Baker T.B., Bailey W.C., Benowitz N.L., Curry S.J. (2008). Treating tobacco use and dependence: 2008 update U.S. public health service clinical practice guideline executive summary. Respir. Care.

[4] Cummings K.M., Fix B.V., Celestino P., Hyland A., Mahoney M., Ossip D.J. (2010). Does the number of free nicotine patches given to smokers calling a quitline influence quit rates: results from a quasi-experimental study. BMC Publ. Health.

[5] Zhu S.H., Stretch V., Balabanis M., Rosbrook B., Sadler G., Pierce J.P. (1996). Telephone counseling for smoking cessation: effects of single-session and multiple-session interventions. J. Consult. Clin. Psychol..

[6] Stead L.F., Perera R., Lancaster T. (2006). Telephone counselling for smoking cessation. Cochrane Database Syst. Rev..

[7] An L.C., Schillo B.A., Kavanaugh A.M., Lachter R.B., Luxenberg M.G., Wendling A.H. (2006). Increased reach and effectiveness of a statewide tobacco quitline after the addition of access to free nicotine replacement therapy. Tobac. Contr..

[8] Bush T., Levine M.D., Deprey M., Cerutti B., Zbikowski S.M., McAfee T. (2008). Prevalence of weight concerns and obesity among smokers calling a quitline. J. Smok. Cessat..

[9] Swartz S.H., Cowan T.M., Klayman J.E., Welton M.T., Leonard B.A. (2005). Use and effectiveness of tobacco telephone counseling and nicotine therapy in maine. Am. J. Prev. Med..

[10] Smith S.S., Keller P.A., Kobinsky K.H., Baker T.B., Fraser D.L., Bush T. (2013). Enhancing tobacco quitline effectiveness: identifying a superior pharmacotherapy adjuvant. Nicotine & tobacco research: official. J. Soc. Res. Nicotine Tobac..

[11] Clark M.M., Hurt R.D., Croghan I.T., Patten C.A., Novotny P., Sloan J.A. (2006). The prevalence of weight concerns in a smoking abstinence clinical trial. Addict. Behav..

[12] Meyers A.W., Klesges R.C., Winders S.E., Ward K.D., Peterson B.A., Eck L.H. (1997). Are weight concerns predictive of smoking cessation? A prospective analysis. J. Consult. Clin. Psychol..

[13] Borrelli B., Mermelstein R. (1998). The role of weight concern and self-efficacy in smoking cessation and weight gain among smokers in a clinic-based cessation program. Addict. Behav..

[14] French S.A., Jeffery R.W., Pirie P.L., McBride C.M. (1992). Do weight concerns hinder smoking cessation efforts?. Addict. Behav..

[15] Aubin H.J., Farley A., Lycett D., Lahmek P., Aveyard P. (2012). Weight gain in smokers after quitting cigarettes: meta-analysis. BMJ (Clinical Research Ed).

[16] Farley A.C., Hajek P., Lycett D., Aveyard P. (2012). Interventions for preventing weight gain after smoking cessation. Cochrane Database Syst. Rev..

[17] Aveyard P., Lycett D., Farley A. (2012). Managing smoking cessationrelated weight gain. Pol. Arch. Med. Wewn..

[18] Bush T.M., Levine M.D., Magnusson B., Cheng Y., Chen X., Mahoney L. (2014). Impact of baseline weight on smoking cessation and weight gain in quitlines. Ann. Behav. Med. A Publ. Soc. Behav. Med..

[19] Chiolero A., Faeh D., Paccaud F., Cornuz J. (2008). Consequences of smoking for body weight, body fat distribution, and insulin resistance. Am. J. Clin. Nutr..

[20] Scherr A., Seifert B., Kuster M., Meyer A., Fagerstroem K.O., Tamm M. (2015). Predictors of marked weight gain in a population of health care and industrial workers following smoking cessation. BMC Publ. Health.

[21] Spring B., Howe D., Berendsen M., McFadden H.G., Hitchcock K., Rademaker A.W. (2009). Behavioral intervention to promote smoking cessation and prevent weight gain: a systematic review and meta-analysis. Addiction (Abingdon, England).

[22] Parsons A.C., Shraim M., Inglis J., Aveyard P., Hajek P. (2009). Interventions for preventing weight gain after smoking cessation. Cochrane Database Syst. Rev..

[23] Copeland A.L., Martin P.D., Geiselman P.J., Rash C.J., Kendzor D.E. (2006). Smoking cessation for weight-concerned women: group vs. individually tailored, dietary, and weight-control follow-up sessions. Addict. Behav..

[24] Bush T., Lovejoy J.C., Deprey M., Carpenter K.M. (2016). The effect of tobacco cessation on weight gain, obesity, and diabetes risk. Obesity Silver Spring, Md.

[25] Spring B., Pagoto S., Pingitore R., Doran N., Schneider K., Hedeker D. (2004). Randomized controlled trial for behavioral smoking and weight control treatment: effect of concurrent versus sequential intervention. J. Consult. Clin. Psychol..

[26] Bush T., Lovejoy J., Javitz H., Magnusson B., Torres A.J., Mahuna S. (2016). Comparative effectiveness of adding weight control simultaneously or sequentially to smoking cessation quitlines: study protocol of a randomized controlled trial. BMC Publ. Health.

[27] Campbell H.S., Ossip-Klein D., Bailey L., Saul J. (2007). Minimal dataset for quitlines: a best practice. Tobac. Control.

[28] Kroenke K., Spitzer R.L., Williams J.B. (2003). The patient health questionnaire-2: validity of a two-item depression screener. Med. Care.

[29] Hollis J.F., McAfee T.A., Fellows J.L., Zbikowski S.M., Stark M., Riedlinger K. (2007). The effectiveness and cost effectiveness of telephone counselling and the nicotine patch in a state tobacco quitline. Tobac. Control.

[30] Zbikowski S.M., Jack L.M., McClure J.B., Deprey M., Javitz H.S., McAfee T.A. (2011). Utilization of services in a randomized trial testing phone- and web-based interventions for smoking cessation. Nicotine Tob. Res. Offic. J. Soc. Res. Nicotine Tob..

[31] Bricker J.B., Bush T., Zbikowski S.M., Mercer L.D., Heffner J.L. (2014). Randomized trial of telephone-delivered acceptance and commitment therapy versus cognitive behavioral therapy for smoking cessation: a pilot study. Nicotine Tob. Res. Offic. J. Soc. Res. Nicotine Tob..

[32] Bush T., Zbikowski S.M., Mahoney L., Deprey M., Mowery P., Cerutti B. (2012). State quitlines and cessation patterns among adults with selected chronic diseases in 15 states, 2005-2008. Prev. Chronic Dis..

[33] Bush T., Levine M.D., Beebe L.A., Cerutti B., Deprey M., McAfee T. (2012). Addressing weight gain in smoking cessation treatment: a randomized controlled trial. Am. J. health Promot. AJHP.

[34] Levine M.D., Bush T., Magnusson B., Cheng Y., Chen X. (2013). Smoking-related weight concerns and obesity: differences among normal weight, overweight, and obese smokers using a telephone tobacco quitline. Nicotine Tob. Res. Offic. J. Soc. Res. Nicotine Tob..

[35] Niedhammer I., Bugel I., Bonenfant S., Goldberg M., Leclerc A. (2000). Validity of self-reported weight and height in the French GAZEL cohort. Int. J. Obes. Relat. Metabolic Disord.: J. Int. Assoc. Study Obes..

[36] Nyholm M., Gullberg B., Merlo J., Lundqvist-Persson C., Rastam L., Lindblad U. (2007). The validity of obesity based on self-reported weight and height: implications for population studies. Obesity Silver Spring, Md.

